# Enteric Duplication Cyst Leading to a Volvulus in an Eight-Month-Old: A Case Report

**DOI:** 10.7759/cureus.78376

**Published:** 2025-02-02

**Authors:** Mounir Salek, Zakaria Chahbi, Fatimazohra E Yaagoubi, Mohamed Ayez, Mohamed El Jdid, Achraf Miry, Sanae Abbaoui, Soukaina Wakrim

**Affiliations:** 1 Radiology, Souss Massa University Hospital, Ibn Zohr University, Agadir, MAR; 2 Radiology, Hospices Civils de Lyon, Lyon, FRA; 3 Pediatric Surgery, Hassan II Regional Hospital, Agadir, MAR; 4 Pathology, Souss Massa University Hospital, Ibn Zohr University, Agadir, MAR

**Keywords:** abdominal imaging, emergency surgery, enteric duplication cyst, pediatric intestinal obstruction, whirlpool sign

## Abstract

Enteric duplication cysts are rare congenital anomalies of the gastrointestinal tract. They may remain asymptomatic or present with non-specific symptoms such as vomiting, abdominal distension, and intestinal obstruction. We report the case of an eight-month-old male infant with no significant medical history who presented with a four-day history of vomiting that became bilious, accompanied by generalized hypotonia and dehydration. Clinical evaluation revealed a Glasgow Coma Scale of 11/15 and mild hyponatremia with otherwise normal laboratory results. Abdominal ultrasound showed inversion of the superior mesenteric artery and vein, raising suspicion for malrotation. Contrast-enhanced CT confirmed the "whirlpool sign" and identified a 50 mm enteric duplication cyst. Emergency surgery revealed a volvulus caused by the cyst, which was surgically corrected with successful resection. The postoperative course was uneventful. This case underscores the importance of early imaging and prompt surgical intervention in infants with bilious vomiting to prevent severe complications from rare conditions like enteric duplication cysts.

## Introduction

Enteric duplication cysts are rare congenital anomalies of the gastrointestinal tract, with an estimated incidence of approximately one in 4,500 live births [1]. These cysts typically present during early childhood and may remain asymptomatic or manifest with non-specific clinical symptoms such as vomiting, abdominal distension, and signs of intestinal obstruction [2]. In the presented case, an eight-month-old male infant exhibited bilious vomiting and generalized hypotonia, subsequently diagnosed with an enteric duplication cyst complicated by midgut volvulus. This case highlights the diagnostic complexity and the critical need for timely intervention in managing enteric duplication cysts in pediatric patients to prevent life-threatening complications.

## Case presentation

An eight-month-old male infant with no significant medical history was admitted to the hospital with a four-day history of vomiting that was initially non-bilious but became bilious one day prior to presentation. On examination, he appeared generally hypotonic with clinical signs of dehydration and had a Glasgow Coma Scale (GCS) score of 11 out of 15. Laboratory investigations revealed mild hyponatremia and a markedly elevated C-reactive protein (CRP) level of 149 mg/L (normal range: <6 mg/L), suggesting an underlying inflammatory process. The complete blood count was normal, renal function was within normal limits, and other electrolyte levels were normal.

**Figure 1:**
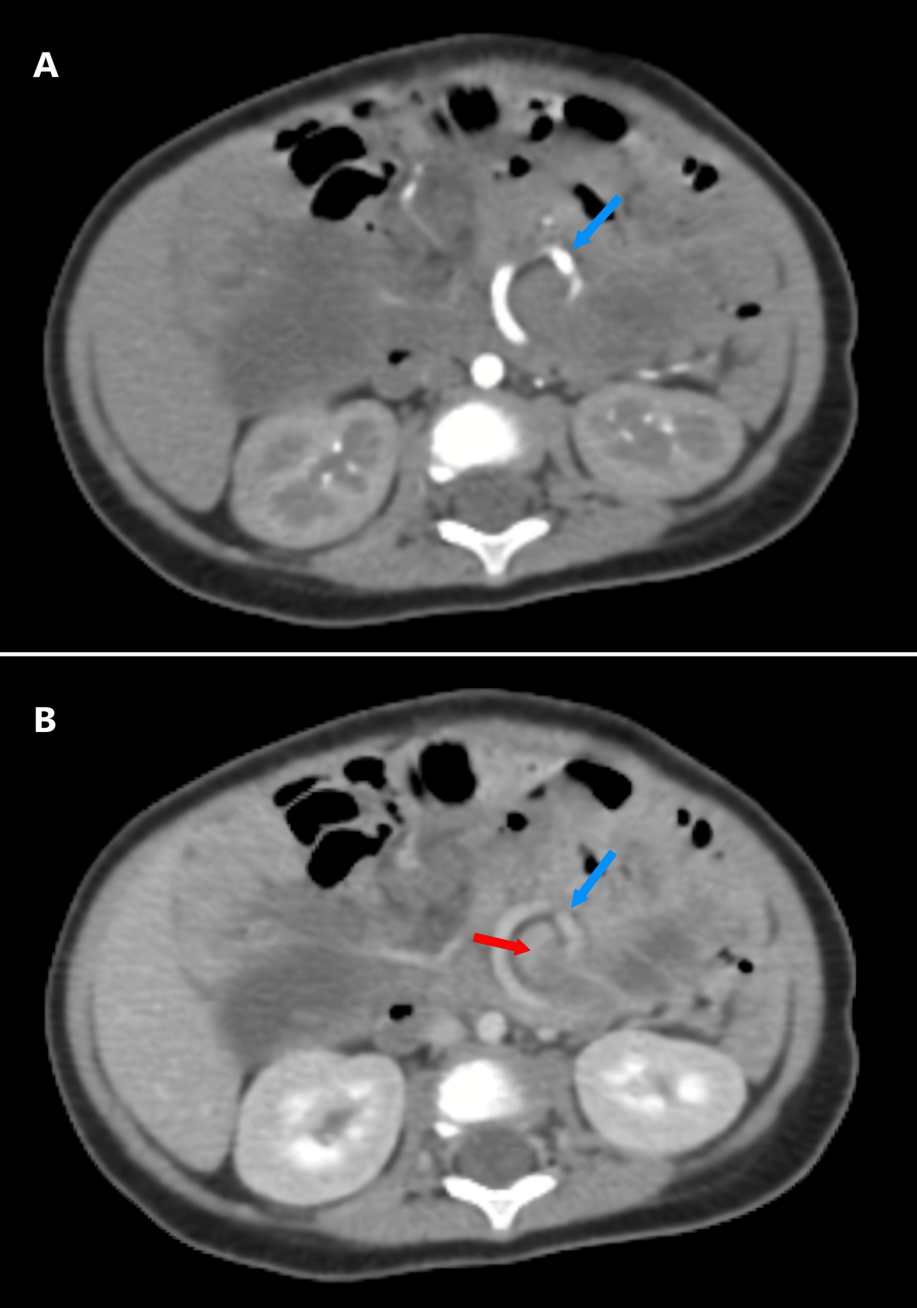
Axial CT scan findings A: arterial phase; B: venous phase showing the “whirlpool sign” pathognomonic of mesenteric volvulus (red arrow: twisted intestinal loop, blue arrow: mesenteric vessels).

**Figure 2:**
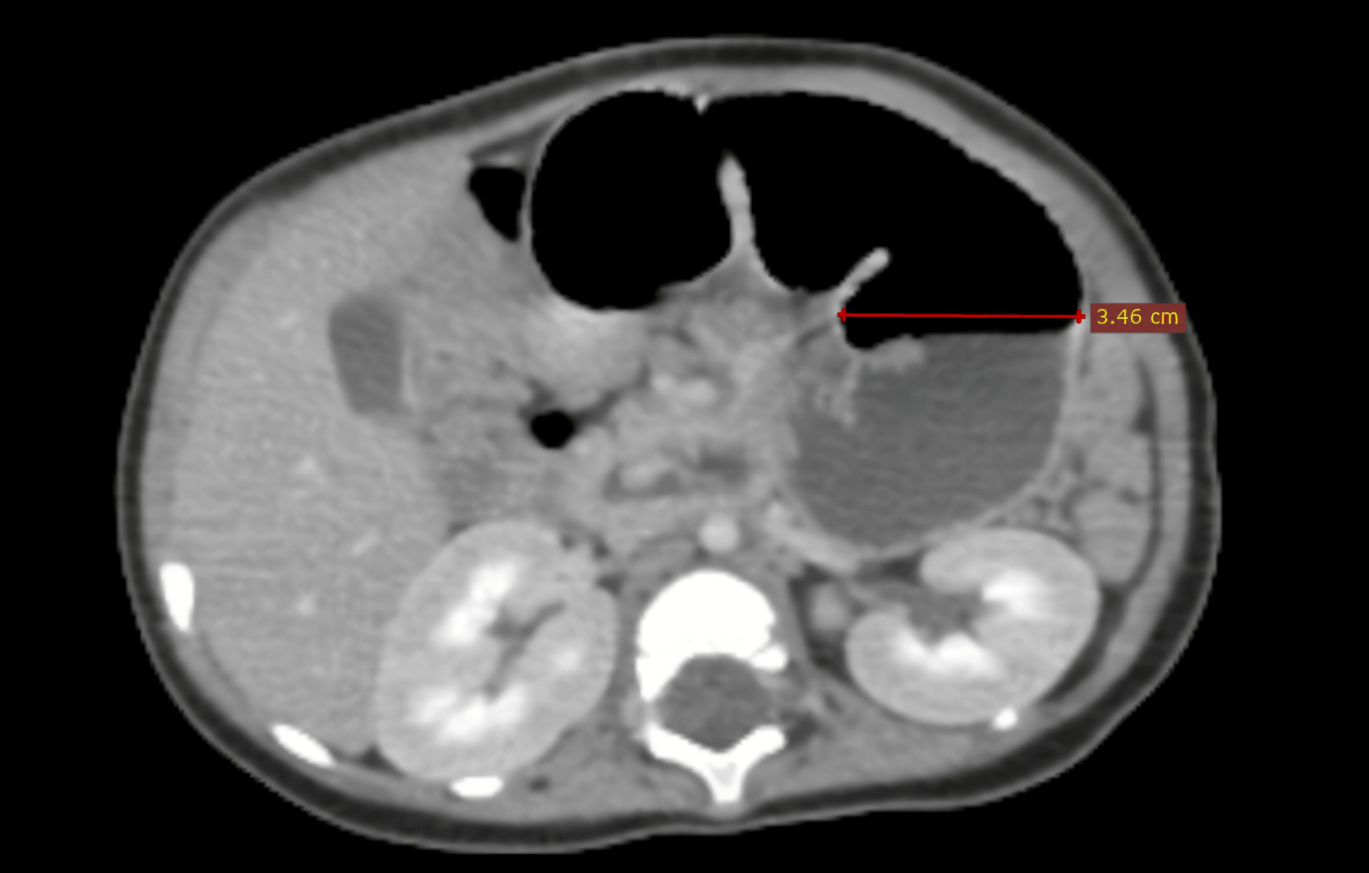
Axial CT image showing the distended loop prior to the obstruction transition zone

**Figure 3:**
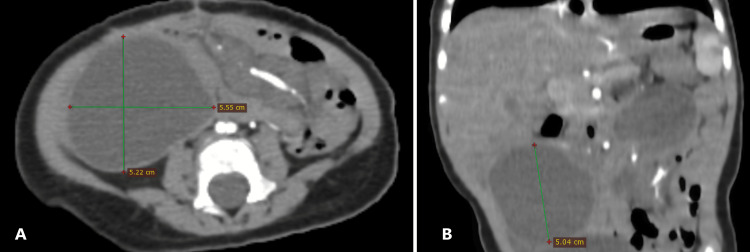
CT scan findings A: axial; B: coronal images of CT showing the enteric duplication cyst adjacent to the volvulus zone.

An abdominal ultrasound revealed an inversion of the superior mesenteric artery (SMA) and vein (SMV), multiple lymph nodes along the mesenteric vessel axis, and a compressed intestinal loop. Further evaluation with a contrast-enhanced computed tomography (CT) scan in arterial and venous phases confirmed the presence of the characteristic "whirlpool sign" (Figure 1), indicating twisted mesenteric vessels around the SMA axis. Additionally, a notably distended loop of the bowel measuring up to 34 mm in diameter was identified (Figure 2). Downstream from this transition point, imaging revealed a well-circumscribed round cystic lesion approximately 50 mm in diameter (Figure 3), consistent with an enteric duplication cyst. The bowel walls displayed normal enhancement with no signs of ischemia, and a small amount of peritoneal fluid was observed in the right iliac fossa.

**Figure 4:**
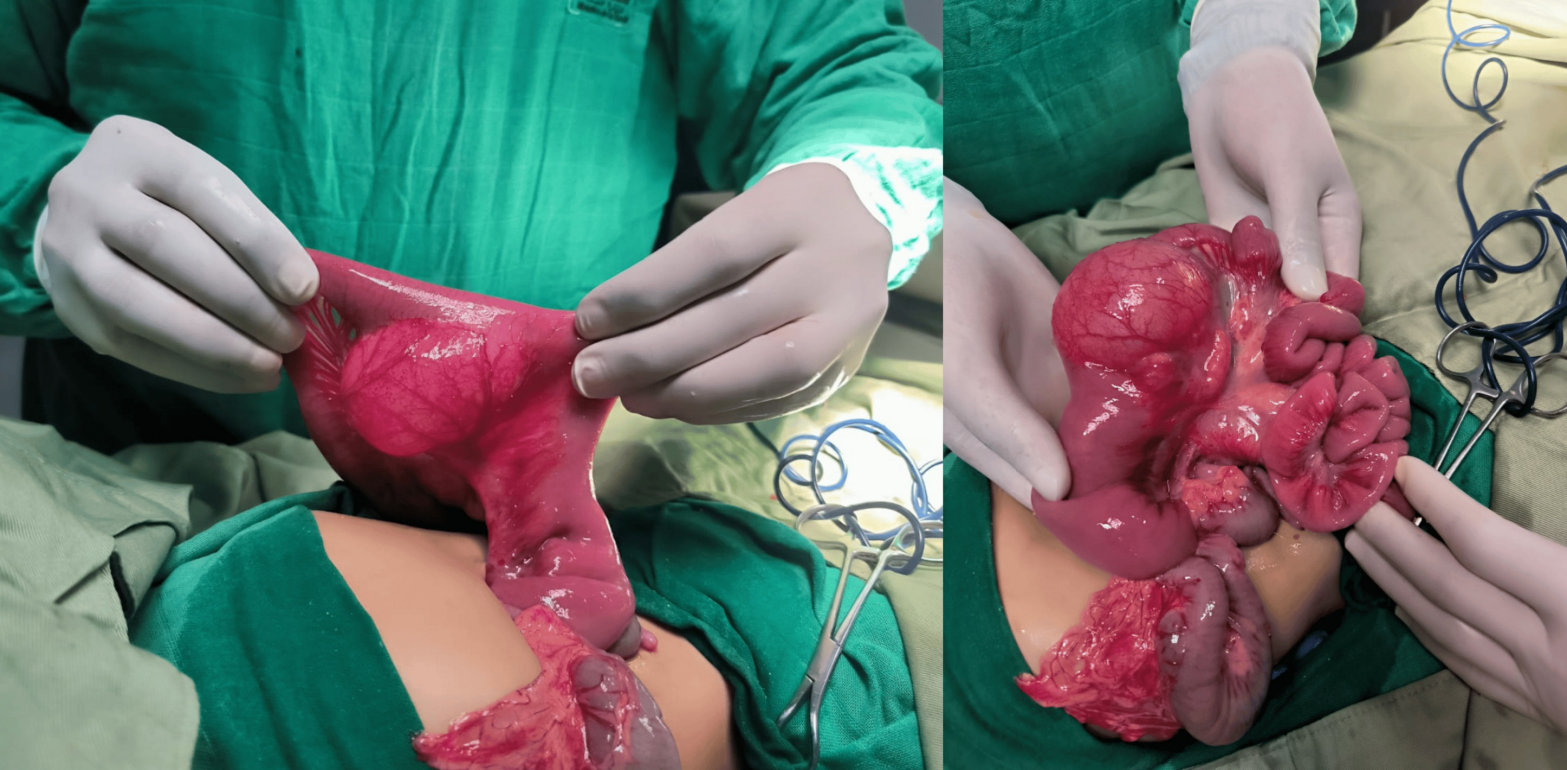
Peroperative images showing the enteric duplication cyst

The patient was stabilized with parenteral rehydration and promptly taken to surgery. Intraoperative findings confirmed a volvulus caused by an enteric duplication cyst (Figure 4), around which the small bowel had twisted. Surgical management involved detorsion of the volvulus and resection of the duplication cyst. The postoperative period was uneventful, with the patient receiving supportive care and close monitoring. Follow-up assessments indicated a favorable recovery and no immediate complications were reported.

**Figure 5:**
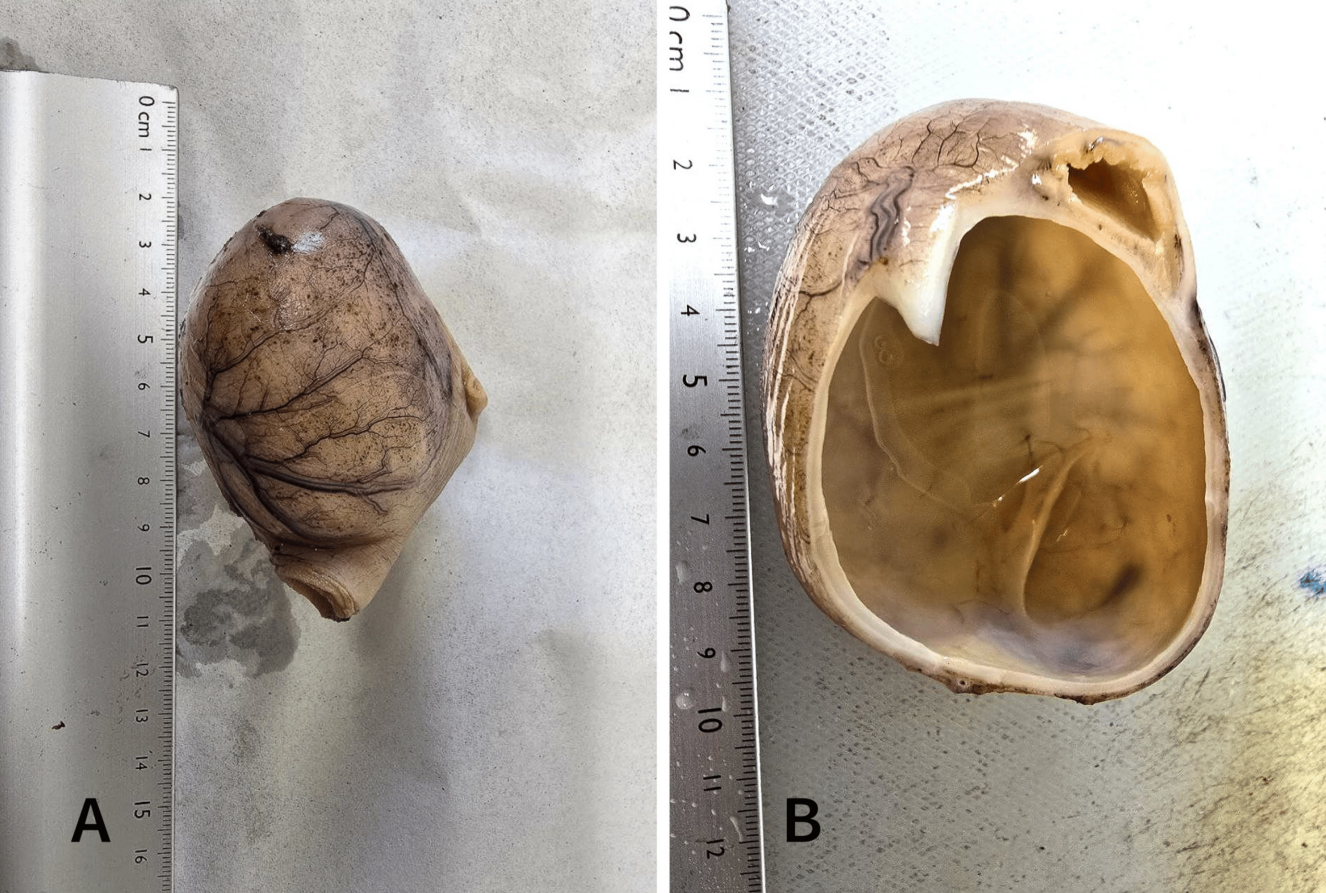
Histological examination findings A: image showing a segment of the small intestine attached to a cystic formation measuring 5.5 cm at its largest axis; B: the cystic formation has a thin wall and a yellowish fluid content.

**Figure 6:**
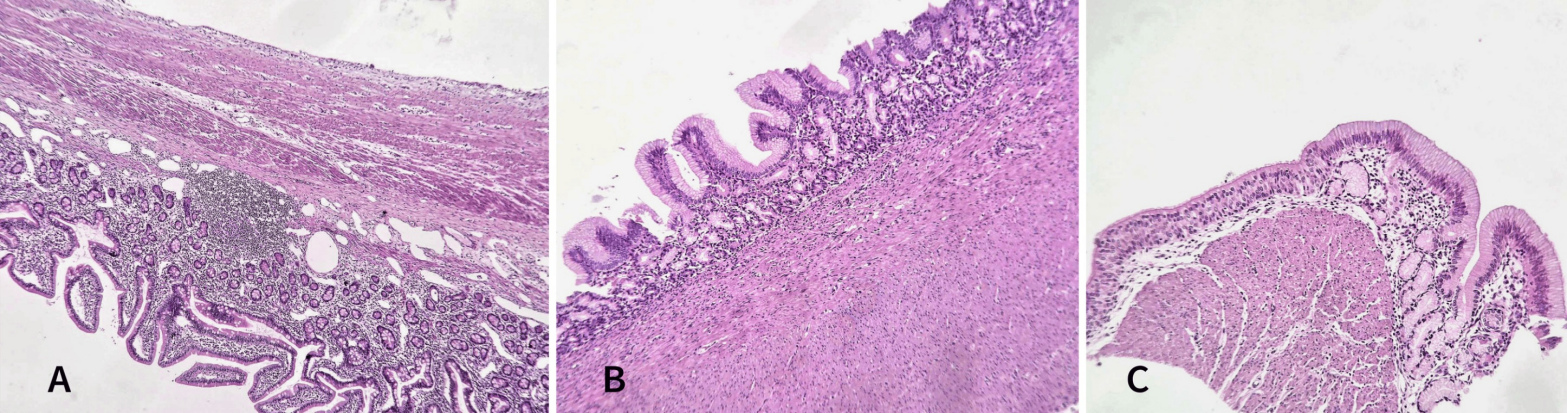
Microphotograph findings A: microphotograph revealing a cystic wall sharing a proper muscular layer with that of the ileal wall (the stain used is hematoxylin and eosin (HE), magnification x100); B: the cystic wall focally exhibits a complete digestive-type wall with gastric-type mucosa (the stain used is hematoxylin and eosin (HE), magnification x200); C: other regions of the cyst contain ciliated respiratory mucosa (the stain used is hematoxylin and eosin (HE), magnification x400).

Histological examination revealed findings consistent with an enteric duplication cyst (Figure 5). The intestinal wall showed features of ileal-type mucosa with well-preserved villi covered by a regular simple columnar epithelium. The lamina propria contained sparse inflammatory cells, primarily lymphocytes, and plasma cells, without neutrophils or eosinophils. Lymphoid follicles were present, and the glands of Lieberkühn appeared normal. The wall of the cystic lesion alternated between complete intestinal layers, including mucosa, submucosa, muscularis propria, and subserosa, and incomplete layers comprising only mucosa. The mucosa was variably of gastric type (antro-fundic) with crypts and antral glands containing some parietal cells or ciliated respiratory epithelium (Figure 6). Focal abrasion of the mucosa was noted. No evidence of malignant tumor proliferation was identified.

## Discussion

Enteric duplication cysts (EDCs) are congenital anomalies that can occur anywhere along the gastrointestinal tract but are most frequently found in the ileum, followed by the jejunum and stomach [2]. Their clinical manifestations vary depending on the size, location, and presence of complications. In this patient, the progression from non-bilious to bilious vomiting was a critical indicator of a possible mechanical obstruction, necessitating urgent evaluation. This is consistent with reports that bilious vomiting in neonates and infants should prompt immediate investigation for conditions like malrotation with volvulus [3].​

Imaging plays a pivotal role in diagnosing enteric duplication cysts. Ultrasound (US) is typically the first-line imaging modality due to its safety and effectiveness. The "double-wall sign" or the presence of a cystic structure with an inner echogenic mucosal layer and outer hypoechoic muscle layer is characteristic of duplication cysts​ [2]. However, in this case, ultrasound findings revealed an inversion of the superior mesenteric artery (SMA) and vein (SMV), raising suspicion for malrotation or volvulus. This necessitated further imaging with contrast-enhanced computed tomography (CT), which revealed the classic "whirlpool sign," confirming the volvulus secondary to the duplication cyst. Studies have shown that CT can effectively delineate vascular anatomy and bowel involvement, particularly when US findings are inconclusive ​[4].

The association between enteric duplication cysts and volvulus is rare but has been documented in several case reports​​ [3,5]. The mass effect, or abnormal attachment of the cyst, can distort mesenteric anatomy, creating a focal point for bowel rotation. In this case, the cyst's proximity to the mesenteric vessels likely predisposed the patient to volvulus, as evidenced by the imaging findings and intraoperative confirmation. Similar cases have highlighted the importance of considering enteric duplication cysts in the differential diagnosis of volvulus, especially when imaging findings are atypical for classic malrotation​ [5].

Surgical intervention remains the definitive treatment for symptomatic or complicated enteric duplication cysts. The standard approach involves cyst excision along with the involved bowel segment to prevent recurrence and address complications such as volvulus, bleeding, or perforation [1]. In this case, the patient underwent emergency surgery, where the volvulus was corrected, and the duplication cyst was resected. Timely surgical intervention is crucial, as delays can result in bowel ischemia or necrosis, significantly increasing morbidity and mortality​ [6].

With the increased use of antenatal imaging, EDCs are now frequently identified before birth. Although the ideal timing for surgical resection remains uncertain, prompt evaluation followed by surgery within the first six months of life is generally recommended [1,7,8].

The prognosis for patients undergoing timely surgical correction of enteric duplication cysts complicated by volvulus is generally favorable. Postoperative recovery is typically uneventful if the bowel remains viable and the cyst is completely excised. In this case, the patient's early postoperative course was favorable, reflecting outcomes reported in similar cases [9].

## Conclusions

This case emphasizes the need for heightened clinical suspicion and prompt imaging in infants presenting with bilious vomiting. While malrotation with volvulus is a common cause, rarer conditions such as enteric duplication cysts should be considered, especially when imaging reveals atypical findings. Early diagnosis and surgical intervention are vital to preventing serious complications and ensuring favorable outcomes. This case contributes to the growing evidence that enteric duplication cysts, though rare, can lead to life-threatening complications like volvulus and must be promptly recognized and managed.
